# Applying a Smartwatch to Predict Work-related Fatigue for Emergency Healthcare Professionals: Machine Learning Method

**DOI:** 10.5811/westjem.58139

**Published:** 2023-07-07

**Authors:** Sot Shih-Hung Liu, Cheng-Jiun Ma, Fan-Ya Chou, Michelle Yuan-Chiao Cheng, Chih-Hung Wang, Chu-Lin Tsai, Wei-Jou Duh, Chien-Hua Huang, Feipei Lai, Tsung-Chien Lu

**Affiliations:** * National Taiwan University Hospital, Department of Emergency Medicine, Taipei, Taiwan; † National Taiwan University, Graduate Institute of Biomedical Electronics and Bioinformatics, Taipei, Taiwan; ‡ MOST Joint Research Center for AI Technology and All VISTA Healthcare (AINTU), Taipei, Taiwan; § National Taiwan University, Department of Emergency Medicine, College of Medicine, Taipei, Taiwan; || National Taiwan University, Department of Computer Science and Information Engineering, Taipei, Taiwan

## Abstract

**Introduction:**

Healthcare professionals frequently experience work-related fatigue, which may jeopardize their health and put patient safety at risk. In this study, we applied a machine learning (ML) approach based on data collected from a smartwatch to construct prediction models of work-related fatigue for emergency clinicians.

**Methods:**

We conducted this prospective study at the emergency department (ED) of a tertiary teaching hospital from March 10–June 20, 2021, where we recruited physicians, nurses, and nurse practitioners. All participants wore a commercially available smartwatch capable of measuring various physiological data during the experiment. Participants completed the Multidimensional Fatigue Inventory (MFI) web form before and after each of their work shifts. We calculated and labeled the before-and-after-shift score differences between each pair of scores. Using several tree-based algorithms, we constructed the prediction models based on features collected from the smartwatch. Records were split into training/validation and testing sets at a 70:30 ratio, and we evaluated the performances using the area under the curve (AUC) measure of receiver operating characteristic on the test set.

**Results:**

In total, 110 participants were included in this study, contributing to a set of 1,542 effective records. Of these records, 85 (5.5%) were labeled as having work-related fatigue when setting the MFI difference between two standard deviations as the threshold. The mean age of the participants was 29.6. Most of the records were collected from nurses (87.7%) and females (77.5%). We selected a union of 31 features to construct the models. For total participants, CatBoost classifier achieved the best performances of AUC (0.838, 95% confidence interval [CI] 0.742–0.918) to identify work-related fatigue. By focusing on a subgroup of nurses <35 years in age, XGBoost classifier obtained excellent performance of AUC (0.928, 95% CI 0.839–0.991) on the test set.

**Conclusion:**

By using features derived from a smartwatch, we successfully built ML models capable of classifying the risk of work-related fatigue in the ED. By collecting more data to optimize the models, it should be possible to use smartwatch-based ML models in the future to predict work-related fatigue and adopt preventive measures for emergency clinicians.

## INTRODUCTION

Work-related fatigue is a major concern in the workplace. Work-related fatigue among medical personnel can affect the health and well-being of emergency healthcare professionals (EHP) and put patient safety at risk.^1^–^6^ Causes of fatigue in the workplace may be due to either physiological factors, such as long work shifts or heavy workload, or psychological aspects such as stress.^2^,^7^ Researchers found that if employees worked more than 55 hours per week, the chances of getting a stroke or coronary heart disease would increase by 33% and 13%, respectively, compared to employees whose working hours met the 55-hour standard.^8^ Another study showed that doctors in Taiwan might have greater occupational pressure and a higher depression rate (13.3%) than that found in the general population (3.7%).^9^ To reduce healthcare workload and prevent human errors during clinical practice by determining how to detect work-related fatigue earlier and adopt preventive measures is worthy of careful study.

Numerous studies have focused on work-related fatigue; however, most were conducted subjectively by using self-report measures such as questionnaires, surveys, or rating scales completed after work.^10^–^12^ Until now, few studies have looked at objective ways of collecting real-time data from EPs and other healthcare workers while on shift. With the advancement of information technology and the advent of wearable devices capable of unobtrusively collecting real-time biosensor data without affecting the workflow of medical staff, it is possible to develop a smartwatch-based prediction model of work-related fatigue for EHPs. In the past, wearable devices focused more on health monitoring for the elderly or fall detection in patients with movement disorders.^13^ Now, wearable devices are used with machine learning (ML) to determine whether a patient has depression or to facilitate the delivery of high-quality cardiopulmonary resuscitation.^14^,^15^

A recent review article by Martins et al focused on fatigue monitoring through wearables. They retrieved a total of 612 articles in their literature search and included 60 articles for analysis. Of the included research, the most common studies were related to drowsiness detection for drivers (33), followed by the detection of physical fatigue, mental fatigue, muscle fatigue and, lastly, vigilance detection. Although four studies focused on the healthcare domain, none looked at healthcare professionals. Three of those studies were related to fatigue detection for rehabilitation patients, and one examined mental fatigue detection in a healthy population. Of the 60 studies included in the review by Martins et al, only five reported on the use of a smartwatch as the signal source for fatigue detection.^16^

Apart from that review, one study described the use of a wearable photoplethysmography (PPG) biosensor to evaluate the feasibility of collecting and analyzing PPG data for burnout in EPs while they work. Since this study showed no significant changes in pulse rate and pulse rate variability over the course of an EP’s academic year, it suggested that alternative methods would have to be explored to measure stress among EPs at work.^17^

**Population Health Research Capsule**

What do we already know about this issue?

*Emergency healthcare professionals frequently experience work-related fatigue, which can impact their health and put patients at risk*.

What was the research question?

*Can machine learning based on smartwatch data predict work-related fatigue in emergency healthcare professionals?*

What was the major finding of the study?

*CatBoost classifier achieved an area under the curve of 0.838 (95% CI 0.742–0.918) for identifying work-related fatigue in study participants*.

How does this improve population health?

*By using smartwatch data and machine learning, work-related fatigue can potentially be identified and prevented, improving patient safety and healthcare professionals’ health*.

A smartwatch is a wearable device with the function of both telling time and acting as a source for collecting physiologic data derived directly from the wearer’s body because of its proximity to the skin. Today most of the commercially available smartwatches claim to have the ability to track the wearer’s health by providing data, such as number of footsteps, heart rate, blood pressure, and oximetry via the embedded biosensors. Although the accuracy of the measured data derived from smartwatches varies depending on the device manufacturer, most mainstream devices can reliably measure heart rate, steps, and other health evaluation indicators.^18^

It is reasonable to conclude that we can collect real-time physiologic data transmitted from the smartwatch worn by healthcare workers to build a work-related fatigue prediction model. By combining objective data collected from a wearable device with the subjective results of the well-validated Multidimensional Fatigue Inventory (MFI) (filled out by the participants as a tool for fatigue labeling),^19^ we sought to apply ML approaches to construct a work-related fatigue prediction model for emergency healthcare professionals. We hypothesized that a smartwatch-based ML model could serve as a reliable method to detect fatigue for EHPs working in the emergency department (ED).

## METHODS

### Study Settings

This prospective observational study was conducted March 10–June 20, 2021 at the ED of National Taiwan University Hospital (NTUH), a 2,400-bed, university-affiliated, tertiary teaching hospital with a daily census of ≈300 emergency visits. We recruited emergency care professionals, including EPs, nurses, and nurse practitioners >20 years of age, excluding anyone who could not tolerate wearing a smartwatch. Each participant was provided with an ASUS VivoWatch SP (ASUSTeK Inc, Taipei, Taiwan), a commercially available smartwatch capable of measuring heart rate, blood pressure, oxygen saturation, and footsteps using the embedded sensors. The smartwatch also features calorie consumption, heart rate variability (HRV), and stress index based on proprietary algorithms not shown to the public. Participants were not allowed to remove the smartwatch while they were at work. Nine smartphones (ASUS Zenfone 7) were set up in the ED, acting as gateways to synchronize and transmit data with a private cloud via Bluetooth protocol (Bluetooth SIG, Inc, Kirkland WA). Each phone was paired with 50 smartwatches over the course of the study.

### The Multidimensional Fatigue Inventory

The MFI is a 20-item self-report scale designed to evaluate five dimensions of fatigue: general fatigue; physical fatigue; reduced motivation; reduced activity; and mental fatigue. This instrument was originally developed in the Netherlands for patients with cancer and chronic fatigue and has been translated and validated in several other languages (including Mandarin) for different populations.^19^–^21^ Each dimension contains four items (two items in a positive and two in a negative direction) to be scored on a five-point Likert scale ranging from 1 (yes, that is true) to 5 (no, that is not true). The negative items (items 2, 5, 9, 10, 13, 14, 16, 17, 18, 19) must be recorded before the scores are added up. The summation of the obtainable score ranges from 20 (absence of fatigue) to 100 (maximum fatigue).

For this study, the original MFI scale was translated into a Traditional Chinese version based on previous publications^19^,^20^ (Supplementary Table 1). Participants were asked to complete an MFI web form before and after each of their work shifts. We calculated the differences between each pair of work-shift scores for the subsequent ML task of labeling work-related fatigue. We set the between-score difference of MFI with more than 10, more than one SD, or more than two SDs as positive labels of work-related fatigue. The research method was approved by the institutional review board (NTUH-REC No.: 202011024RIND) of NTUH. All participants signed an informed consent form before participating in the study.

### The Machine-Learning Method

In addition to participants’ demographic data and the time series data of blood pressure, heart rate, HRV, footsteps, and calorie consumption, we retrieved the stress index transmitted from the smartwatches to the private cloud as the main features for constructing ML models. We used a set of time windows (first, second, and fourth hours) and statistical functions (eg, minimum, maximum, mean, slope, SD, etc) to create more features as the input variables. In the feature space we also included the one-hot-encoded (a way to convert variables for use in ML algorithms) demographics of the study participants. Missing data were automatically imputed with dummy variables by the ML models. Initially, 696 features were included as the input variables for the subsequent ML task.

We used several tree-based algorithms—including random forest (RF), gradient boosting (GB), eXtreme gradient boosting (XGBoost) (an open source software library), light gradient boosting machine (LightGBM) (an open source for a distributed gradient-boosting framework for ML developed by Microsoft Corp, Redmond, WA), and categorical boosting (CatBoost) classifiers—to construct the prediction models of work-related fatigue for clinical staff in the ED. We defined the between MFI score differences of more than two SDs in each pair of work shifts as work-related fatigue and used them as the binary classification label for this study. We divided all records into two sets chronologically, 70% of which were assigned to the training and validation set and 30% to the test set. We used k-fold cross-validation during the model training process by setting k from 7 to 10 to obtain the best performances.

We adopted a two-step method as our feature selection strategy. First, we deselected those features with high correlation. Second, we ranked the selected features by using entropy measures based on information gain theory.^22^ Iteratively, we built our models by trying from the top fifth important features and added one more until the last selected features, and finally found a set of features to construct the best models in terms of the performance of area under the curve (AUC) measure of receiver operating characteristic (ROC). Features of continuous data were expressed as mean and SD (or mode and interquartile range depending on the normality test), whereas categorical data were expressed as counts and proportions. We also presented univariate descriptive statistics to evaluate differences between classes by using Student *t*-test, chi-squared test, Fisher exact test, or Mann-Whitney U test depending on the distribution.

To resolve the class imbalance problem for work-related fatigue prediction, we used the synthetic minority oversampling technique method (SMOTE), which oversampled the minority class during the training process.^23^ The selection of the models was based on the AUC performance in the test set, which was set as the primary evaluation metric of the study. For each model we also reported other performance measures, including area under the precision-recall curve (AUPRC), accuracy, negative predictive value (NPV), precision (or positive predictive value [PPV]), recall (or sensitivity, or true positive rate), specificity (true negative rate), kappa, and F-1 score, The entropy measures for feature ranking were performed using Weka 3.8, a collection of ML algorithms for data-mining tasks (University of Waikato, Hamilton, New Zealand).^24^ Other ML analyses were performed using Python 3.8 with the package scikit-learn 0.23.1 installed (Python Software Foundation, Fredericksburg, VA).^25^

## RESULTS

We included 110 participants in this study, with a set of 1,542 effective records collected. Each participant contributed to at least one record, ranging from 1–23 records. Of them, 85 (5.5%) were labeled as having work-related fatigue (two SDs as the threshold) based on our study definition. The mean age of the participants was 29.6 years (SD 6.3), and 77.5% of the records were for females. Most of the collected records were from nurses (87.7%), followed by nurse practitioners (7.7%) and EPs (4.5%) (*P* < 0.001). Up to 47.7% of the collected records were on the evening shift, 44.5% on the day shift, and 7.8% on the night shift (*P* < .001).

The characteristics of the demographic features in the study population are shown in Table 1. The characteristics and univariate analyses of the selected features between participants with or without work-related fatigue are summarized in Supplementary Table 2, shown respectively for the training and testing sets. Based on our feature selection strategy, finally there was a union of 31 features selected to construct the models. Of them, the work start time was the only demographic feature selected. The other 30 features were derived from the smartwatch, of which 11 were related to heart rate, seven to blood pressure, five to stress index, three to HRV, three to calorie assumption, and one related to footsteps.

**Figure 1:**
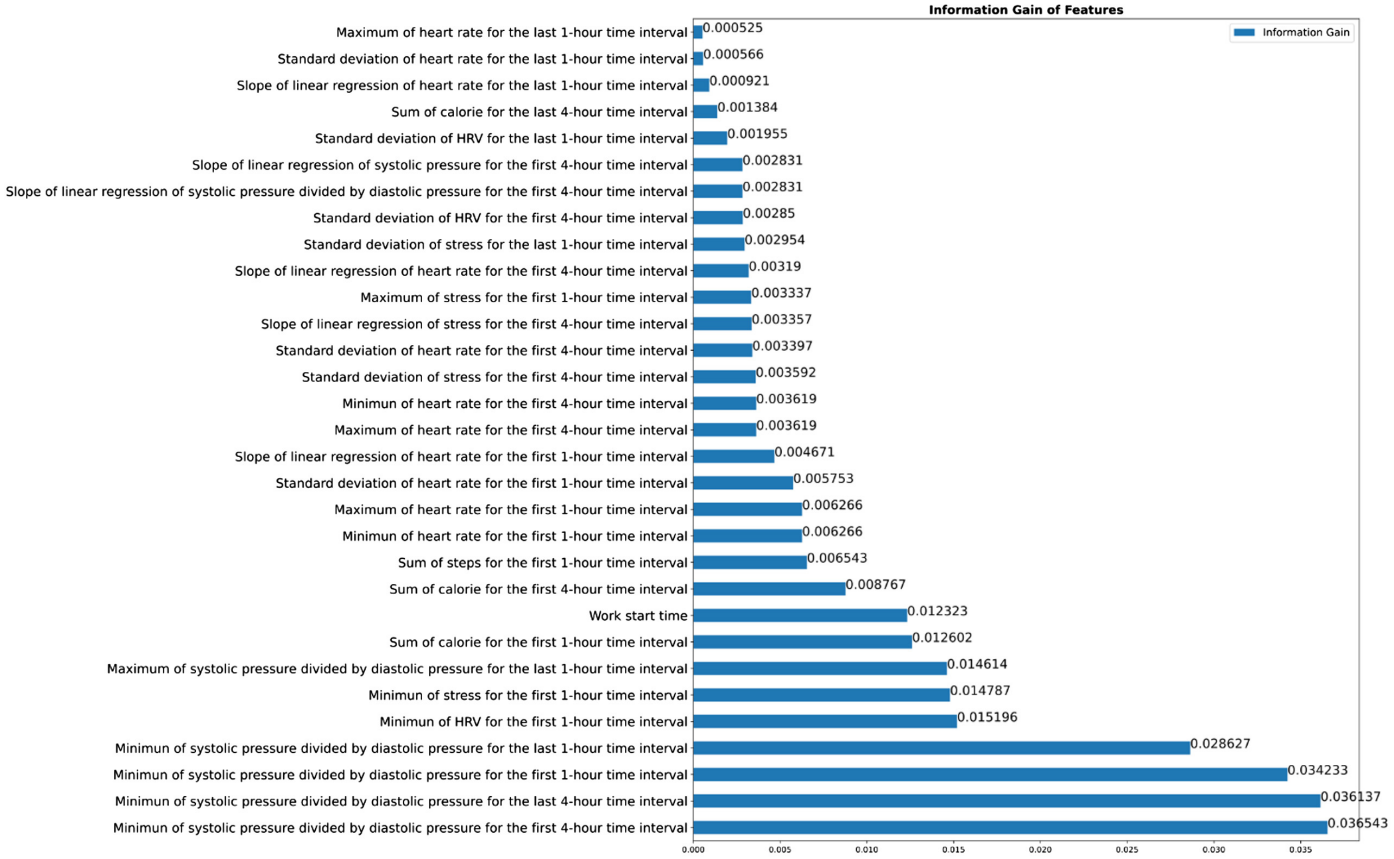
Feature importance ranked by the information gain (entropy). Arranged in an ascending order (from top to bottom), the relatively low information gain of 0.00525 was given by the feature of “maximum of heart rate for the last 1-hour time interval” and the relatively higher information gain of 0.036543 was given by the feature “minimum of systolic pressure divided by diastolic pressure for the first 4-hour time interval.”

The entropy measures for the ranking of the 31 selected features are shown in Figure 1. The initially included 696 features and the percentages of data missing are shown in Supplementary Table 3. The description of the three types of the candidate features (demographics, sensor data from the smartwatch, and statistical data derived from the sensor data) are shown in Supplementary Table 4.

**Figure 2:**
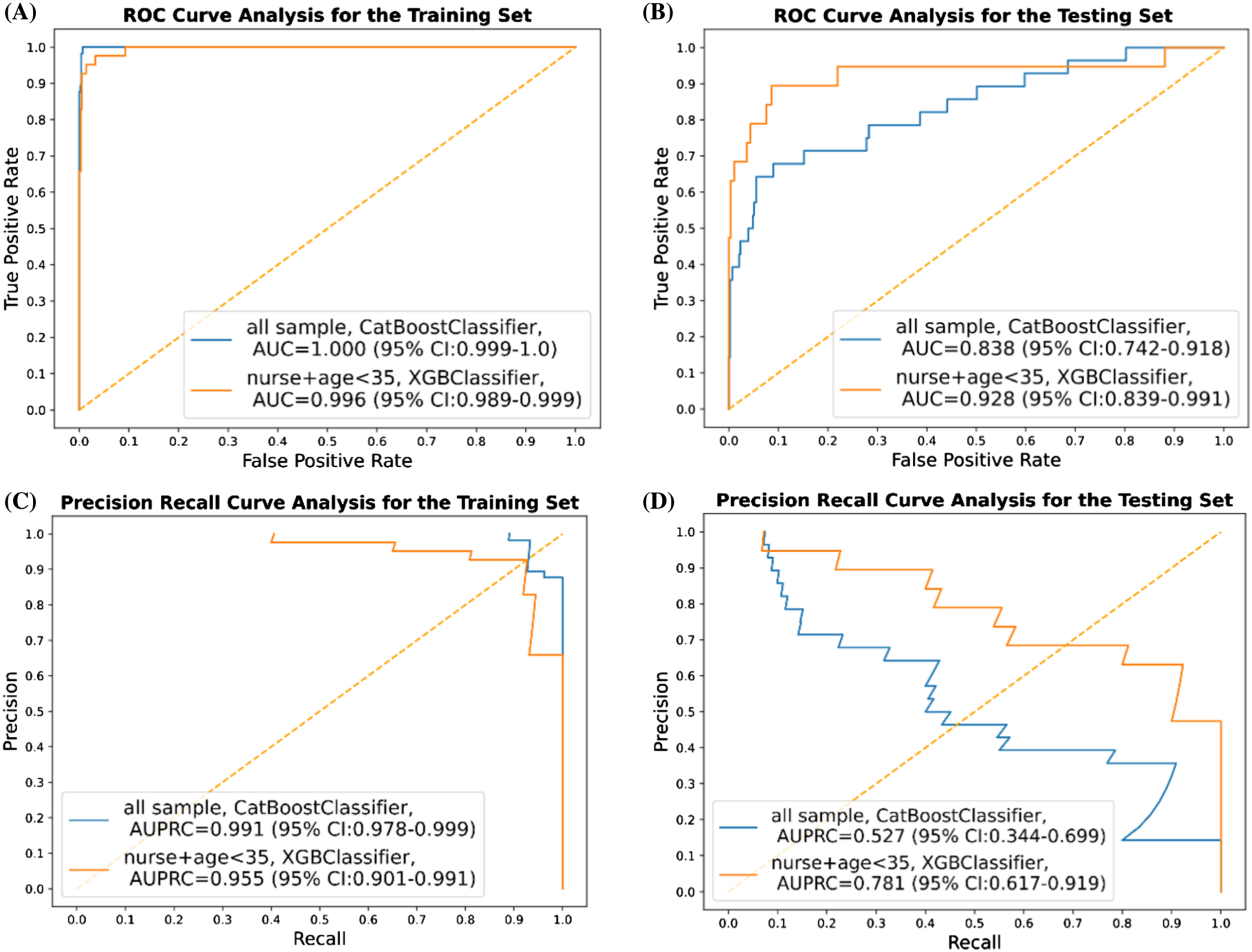
Performance measures of the best machine learning model in terms of AUC and AUPRC for all samples and for subgroup of nurses <35 years in age. (A) AUC on the training cohort (B) AUC on the testing cohort (C) AUPRC on the training cohort (D) AUPRC on the testing cohort. *AUC*, area under the receiver operating characteristic (ROC) curves; *AUPRC*, area under the precision recall curve.

For total participants, the classification results (based on the best AUC in terms of the different thresholds for labeling), including AUC, AUPRC, appa, accuracy, F1-score, precision (PPV), specificity, and NPV on the training and testing sets are presented in Table 2. By adjusting the threshold of work-related fatigue labeling from two SDs to one SD or 20 points of the between differences of the MFI score, here we show merely the top classifier in terms of AUC. By using the CatBoost classifier, the best performances of AUC and AUPRC in the test set were 0.838 (95% confidence interval [CI] 0.742–0.918) and 0.527 (95% CI 0.344–0.699), respectively (Figure 2). Conducting further subgroup analysis when focused on nurses <35 years of age, XGBoost classifier achieved the best performance in terms of AUC of 0.928 (95% CI 0.839–0.991) when setting the threshold for work-related fatigue as two SDs. Meanwhile, other performance measures for this subgroup are presented in Table 3. In this model, AUPRC was 0.781 (95% CI 0.617–0.0.919) (Figure 2).

## DISCUSSION

In this study we applied ML techniques to predict work-related fatigue for clinical staff who worked at the ED in a tertiary hospital in Taiwan. Using 31 selected features derived from a wearable device, we successfully built ML models capable of classifying the risk of work-related fatigue in the ED environment. Instead of using base models like decision tree learning, we used several tree-based algorithms to construct the prediction models. We chose these algorithms for their ability to manage the non-linear relation with better performance in coping with a small amount of data. These algorithms were also less affected by missing values and were independent to feature scale and normalization as well.^26^ While setting the work-related fatigue threshold as two SDs difference between the before-and-after-work MFI scores, we obtained good discriminatory performance to predict work-related fatigue taking AUC as the performance indicator. Of the algorithms we used, CatBoost (AUC 0.838) outperformed RF, GB, XGBoost, and LightGBM classifiers when applied to the testing set for the whole cohort of participants. Since younger participants may have been more likely to have work-related fatigue^27^ and nurses made up the majority of the research group (and their work content might be different from EPs and nurse practitioners), we performed a subgroup analysis by focusing on nurses <35. In this subgroup analysis, the XGBoost classifier (AUC 0.928) outperformed the others on the testing set in this subgroup (Figure 2).

### Feature Selection Using Entropy and Information Gain

Entropy is a measure of the uncertainty or randomness in the data. It is calculated as the sum of the negative of the probability of each class multiplied by the logarithm of the probability of each class. In decision tree algorithms, entropy is used to determine how much information a feature can provide us with in reducing the uncertainty of the target variable.^24^ The entropy is zero when all the instances in a set belong to the same class and is maximum when the instances are equally divided among all classes. Information gain is a measure of how much a particular feature helps in reducing the uncertainty of the target variable. In other words, it tells us how useful a particular feature is in classifying the target variable. Information gain is calculated by subtracting the weighted average entropy of the target variable after the feature is used for classification from the entropy of the target variable before the feature is used. The feature with the highest information gain is chosen as the root node of the decision tree.

We used entropy and information gain as a method of feature selection (instead of the commonly used univariate analysis), given its ability to rank the features in the order of their respective information gains, so that we could select features based on the threshold in the ML algorithms. As shown in Figure 1, we can see the relatively low information gain of 0.00525 given by the feature “maximum of heart rate for the last 1-hour time interval” and the relatively higher information gain of 0.036543 given by the feature “minimum of systolic pressure divided by diastolic pressure for the first 4-hour time interval.” In summary, entropy and information gain could be used to determine the best feature to use for classification, with the goal of reducing the uncertainty of the target variable and maximizing the information gain.

### Comparison with Previous Studies

Work-related fatigue might have both physical and psychological adverse effects on ED clinical staff, which could lead to harmful events both for them and their patients. Until now, previous research studies for work-related fatigue in healthcare practitioners were mostly conducted in the form of a questionnaire survey rather than real-time monitoring.^10^–^12^ With the advancement of wearable devices and the development of artificial intelligence in medicine, novel technology may facilitate real-time identification of work-related fatigue in the healthcare domain. However, previous studies using wearable monitoring methods have focused more on drowsiness detection for drivers’ occupational health, or fatigue detection for rehabilitation patients rather than for healthcare professionals.^16^ With the combination of wearable technology and ML algorithms, these models were considered to perform better than traditional methods of detecting fatigue. However, little was known about the data quality during the phase of model development.^16^ In this study, we successfully constructed ML models based on a smartwatch to identify work-related fatigue, which can serve as a basis to implement a work-related fatigue prediction model for emergency healthcare professionals.

### Interpretation of Current Study

In addition to self-reported symptoms, the autonomic nervous system, especially HRV, was thought to be an indicator related to work-related fatigue.^28^ In our study, of 31 features selected to construct the ML models, three features were related to HRV, 11 to heart rate, seven to blood pressure, five to stress index, three to calorie assumption, and one was related to footsteps.

Our study included 1,542 before-and-after MFI scales completed by participants who wore a smartwatch while on shift from March 10–June 20, 2021. Of the participants, 85 (5.5%) showed work-related fatigue and were judged to have work-related fatigue by our study definition. The training/validation and testing sets were divided chronologically by a ratio of 70:30 to simulate a prospective, randomized controlled trial. Our study achieved good AUC results for all populations and obtained excellent performance in the subgroup analysis for nurses <35 years old.

The rate of work-related fatigue in our population was low (5.5%), which does not coincide with previously reported rates of burnout for EPs. As addressed in two review articles, EPs’ burnout rates were estimated to be 25–60% depending on the study.^29^,^30^ However, most of the reviewed studies focused on research papers that used the Maslach Burnout Inventory (MBI) to assess the prevalence of burnout. Although the MBI is a well-validated tool and addresses three scales (emotional exhaustion, depersonalization, and personal accomplishment), it mainly focuses on the psychological aspect of burnout rather than the five dimensions of fatigue per the MFI, which we used in our study.

In addition to AUC, we evaluated a series of performance measures in our constructed models, including accuracy, NPV, PPV (precision), sensitivity (recall), specificity, kappa, and F1 score (Tables 2 and 3). Precision refers to how often the predictions for positive class are true, while recall refers to how accurately our model can retrieve the relevant records. The class imbalance problem exists in this study. The incidence of work-related fatigue is only 5.5% in our study. Although we obtained an extremely high specificity value (0.828 to 0.989) and NPV (0.831 to 0.962) in the constructed ML models for all populations, our results showed that the sensitivity values (0.354 to 0.457), precision (0.313 to 0.688), and AUPRC (0.354 to 0.527) were relatively low (Table 2). This implies that we must pay more attention to the clinical staff with predicated work-related fatigue, rather than those without.

Due to the excellent performances in specificity and NPV, our ML models may also be used as a tool for identifying clinical staff who are not at risk for work-related fatigue. When such a high specificity tool yields a positive prediction, we can confidently rule in the risk of work-related fatigue for this EP or nurse. When we focused on nurses <35 years old, our prediction models showed some degree of improvement depending on the models we used, which means that different healthcare professionals may have different patterns of work-related fatigue (Table 3).

### Feasibility for Clinical Application

Work-related fatigue would exert both physical and psychological stress on clinical staff, which could further lead to harmful events to healthcare professionals and patients as well. From the previous research, work-related fatigue was mainly reported in a form of a questionnaire survey rather than detection in a timely fashion.^10^–^12^ With our constructed ML model, work-related fatigue could be monitored and documented in real time, and even in the early period of the work shift because many of the 31 selected features were extracted in the first one-hour or four-hour time interval (Figure 1). When clinical staff are thought to have work-related fatigue predicted by our model, we should try to understand their physical and psychological health and initiate risk assessment and preventive measures.

Moreover, we can even adjust and systemize the demand of the workforce according to the frequency of the positive output of work-related fatigue. Work-related fatigue may result in a workforce shortage because of sick leave or resignations.^31^ An insufficient workforce could lead to increasing work-related fatigue due to additional stress from having to share a greater workload or unfamiliarity with the work to be done. To escape the vicious circle, detecting clinical staff who have work-related fatigue and then adjusting workforce demand flexibly is of paramount importance for the medical personnel in the ED and their patients.

## LIMITATIONS

There are limitations to this study. First, we labeled work-related fatigue by using a self-assessment scale, the MFI, which could be subjective to reporting bias. However, the bias may have been minimal since MFI is a well-validated tool worldwide.^20^–^21^ Second, this study was conducted in an ED of a single, tertiary teaching hospital with a small sample size that included only a few overnight workers (7.8%), and focused mostly on nurses who were <35 years in age (the predominant nurse group working in our ED). Selection bias may have been introduced since participants were enrolled voluntarily and not all types of clinical staff in the ED were enrolled. For the generalizability of the models, further study may be required to collect more data from diverse medical staff not included in this study.

Third, because of the low positivity rate of work-related fatigue in our population, our constructed ML models encountered the problem of imbalanced classification and false negatives.^32^ To obtain better performances, we used the SMOTE method during the model training process to balance the dataset. In addition, we evaluated the prediction performances of our models with most of the available performance measures, including AUC, AUPRC, precision, and recall, thereby avoiding over-interpretation by any of the results. Fourth, our models achieved perfect performance (AUC near one) in the training set but obtained only excellent performance in the testing set, and that raised the concern of overfitting. Such overfitting concern can also be seen in the results of high variance (large confidence interval) in the prediction model we built. In our next step, we plan to collect more data and adopt more robust algorithms before our model can be generalizable to the target population.

Fifth, we evaluated only the short-term, work-related fatigue for a shift given that we labeled it by using the between MFI difference as the labeling method. Further study is needed to investigate the method that could measure the longitudinal fatigue, stress, or burnout effects of working in the ED. Finally, due to the policy of intellectual property protection in the consumer electronics industry, the product manufacturer would not disclose any detail related to the data quality. We believe that even though the accuracy of the signal derived from the sensor of the smartwatch is uncertain, the trend and changes of the signal can still serve as the features for building a fatigue-detection model.

## CONCLUSION

We successfully constructed smartwatch-based, machine learning models to predict work-related fatigue with great discrimination ability based on the features monitored by a smartwatch. Implementation of this tool may be useful to identify ED clinical staff at risk of work-related fatigue. Given the small sample size in this study, more data collection and further prospective validation to determine the effectiveness of usage of this prediction model will be necessary before it can be applied in daily clinical practice.

## Supplementary Information









## Figures and Tables

**Table 1:** Characteristics of the demographic features in the study population.

Variables (features)	Total (N = 1,542)	Training cohort (n = 1,079)	Testing cohort (n = 463)	*P* value
**Gender**				.07
**Female**	1,195 (77.5)	850 (78.8)	345 (74.5)	
**Male**	347 (22.5)	229 (21.2)	118 (25.5)	
**Age, mean (SD)**	29.6 (6.3)	29.6 (6.4)	30.5 (3.5)	.84§
**Role**				<.001
**Doctor**	70 (4.5)	38 (3.5)	32 (6.9)	
**Nurse practitioner**	119 (7.7)	104 (9.6)	15 (3.2)	
**Nurse**	1353 (87.7)	937 (86.8)	416 (89.8)	
**Work type**				<.001
**Night shift**	121 (7.8)	34 (3.2)	87 (18.8)	
**Evening shift**	735 (47.7)	519 (48.1)	216 (46.7)	
**Day shift**	686 (44.5)	526 (48.7)	160 (34.6)	
**Work hours, mean (SD)**	8.3 (0.8)	8.2 (0.7)	8.4 (1.0)	<.001
**Seniority**φ, **mean (SD)**	6.3 (5.1)	6.4 (5.1)	5.3 (6.7)	.76
**Day of week**				.012
**Monday**	222 (14.4)	171 (15.8)	51 (11.0)	
**Tuesday**	232 (15.0)	170 (15.8)	62 (13.4)	
**Wednesday**	249 (16.1)	153 (14.2)	96 (20.7)	
**Thursday**	222 (14.4)	153 (14.2)	69 (14.9)	
**Friday**	213 (13.8)	155 (14.4)	58 (12.5)	
**Saturday**	198 (12.8)	135 (12.5)	63 (13.6)	
**Sunday**	206 (13.4)	142 (13.2)	64 (13.8)	
**Work start time**				<.001
**7:00** **am**	60 (3.9)	53 (4.9)	7 (1.5)	
**7:30** **am**	569 (36.9)	437 (40.5)	132 (28.5)	
**8:00** **am**	44 (2.9)	29 (2.7)	15 (3.2)	
**9:00** **am**	13 (0.8)	7 (0.6)	6 (1.3)	
**2:30** **pm**	57 (3.7)	49 (4.5)	8 (1.7)	
**3:30** **pm**	678 (44.0)	470 (43.6)	208 (44.9)	
**8:00** **pm**	26 (1.7)	9 (0.8)	17 (3.7)	
**11:30** **pm**	95 (6.2)	25 (2.3)	70 (15.1)	

§ We used the Student *t*-test as the statistical method to compare the variable age and chi-squared test for remaining variables.

φ Seniority means years of working as a healthcare professional.

**Table 2:** Performance measures for the total participants.

	Total participants					

	Training			Testing		
**Threshold**	2 SDs (=35.8)	1 SD (=17.9)	20	2 SDs (=35.8)	1 SD (=17.9)	20
**Model**	CatBoost	GradientBoosting	XGBoost	CatBoost	GradientBoosting	XGBoost
**Positive count**	57	163	277	28	46	96
**AUC**	1	1	0.996	0.838	0.759	0.647
**AUPRC**	0.991	0.998	0.989	0.527	0.43	0.354
**F1**	0.837	0.979	0.953	0.5	0.372	0.352
**Kappa**	0.829	0.975	0.937	0.477	0.288	0.182
**Sensitivity**	0.719	0.982	0.96	0.393	0.457	0.354
**Speci** **fi** **city**	1	0.996	0.981	0.989	0.89	0.828
**PPV**	1	0.976	0.947	0.688	0.313	0.351
**NPV**	0.985	0.997	0.986	0.962	0.937	0.831
**Accuracy**	0.985	0.994	0.976	0.952	0.847	0.73

*AUC*, area under the receiver operating characteristic curve; *AUPRC*: area under the precision recall curve; *PPV*, positive predictive value; *NPV*, negative predictive value.

**Table 3:** Performance measures for the subgroup of nurses younger than 35 years old.

	Nurses <35 years old					

	Training			Testing		
**Threshold**	2 SDs (=38.2)	1 SD (=19.1)	20	2 SDs (=38.2)	1 SD (=19.1)	20
**Model**	XGBoost	RandomForest	XGBoost	XGBoost	RandomForest	XGBoost
**Positive count**	41	97	174	19	34	66
**AUC**	0.996	0.998	0.928	0.928	0.813	0.738
**AUPRC**	0.955	0.989	0.881	0.781	0.609	0.577
**F1**	0.905	0.926	0.75	0.556	0.429	0.462
**Kappa**	0.899	0.913	0.685	0.515	0.328	0.357
**Sensitivity**	0.927	0.969	0.638	0.789	0.618	0.364
**Speci** **fi** **city**	0.992	0.98	0.979	0.928	0.837	0.939
**PPV**	0.884	0.887	0.91	0.429	0.328	0.632
**NPV**	0.995	0.995	0.889	0.985	0.944	0.838
**Accuracy**	0.988	0.978	0.893	0.919	0.811	0.811

*AUC*, area under the receiver operating characteristic curve; *AUPRC*, area under the precision recall curve; *PPV*, positive predictive value; *NPV*, negative predictive value.
