# Supplementary material for: Applying a Smartwatch to Predict Work-related Fatigue for Emergency Healthcare Professionals: Machine Learning Method
Source: West J Emerg Med. 2023 Jul 7;24(4):693–702. doi: 10.5811/westjem.58139 (PMC10393460; doi:10.5811/westjem.58139)
Supplement: Supplementary file 1 [file wjem-24-693-s001.docx]

**Supplementary Table 1:** The original Multidimensional Fatigue Inventory (MFI) and its Traditional Chinese version.

| **Instruction：**  By means of the following statement we would like to get an idea of how you have been feeling **lately**. There is, for example, the statement:  "I FEEL RELAXED"  If you think that this is **entirely true**, that indeed you have been feeling relaxed lately, please, place an **X** in the extreme left box; like this:  **yes, that is true** ⮽1 □2 □3 □4 □5 **no, that is not true**  The more you disagree with the statement, the more you can place an **X** in the direction of "no, that is not true". Please do not miss out a statement and place only one **X** in a box for each statement. | | | | | | | | |
| --- | --- | --- | --- | --- | --- | --- | --- | --- |
| 01 | I feel fit | yes, that is true | □1 | □2 | □3 | □4 | □5 | no, that is not true |
| 02 | Physically, I feel only able to do a little. | yes, that is true | □1 | □2 | □3 | □4 | □5 | no, that is not true |
| 03 | I feel very active. | yes, that is true | □1 | □2 | □3 | □4 | □5 | no, that is not true |
| 04 | I feel like doing all sorts of nice things. | yes, that is true | □1 | □2 | □3 | □4 | □5 | no, that is not true |
| 05 | I feel tired. | yes, that is true | □1 | □2 | □3 | □4 | □5 | no, that is not true |
| 06 | I think I do a lot in a day. | yes, that is true | □1 | □2 | □3 | □4 | □5 | no, that is not true |
| 07 | When I am doing something, I can keep my thoughts on it. | yes, that is true | □1 | □2 | □3 | □4 | □5 | no, that is not true |
| 08 | Physically, I can take on a lot. | yes, that is true | □1 | □2 | □3 | □4 | □5 | no, that is not true |
| 09 | I dread having to do things. | yes, that is true | □1 | □2 | □3 | □4 | □5 | no, that is not true |
| 10 | I think I do very little in a day. | yes, that is true | □1 | □2 | □3 | □4 | □5 | no, that is not true |
| 11 | I can concentrate well. | yes, that is true | □1 | □2 | □3 | □4 | □5 | no, that is not true |
| 12 | I am rested. | yes, that is true | □1 | □2 | □3 | □4 | □5 | no, that is not true |
| 13 | It takes a lot of effort to concentrate on things | yes, that is true | □1 | □2 | □3 | □4 | □5 | no, that is not true |
| 14 | Physically I feel I am in a bad condition. | yes, that is true | □1 | □2 | □3 | □4 | □5 | no, that is not true |
| 15 | I have a lot of plans. | yes, that is true | □1 | □2 | □3 | □4 | □5 | no, that is not true |
| 16 | I tire easily. | yes, that is true | □1 | □2 | □3 | □4 | □5 | no, that is not true |
| 17 | I get little done. | yes, that is true | □1 | □2 | □3 | □4 | □5 | no, that is not true |
| 18 | I don’t feel like doing anything. | yes, that is true | □1 | □2 | □3 | □4 | □5 | no, that is not true |
| 19 | My thoughts easily wander. | yes, that is true | □1 | □2 | □3 | □4 | □5 | no, that is not true |
| 20 | Physically I feel I am in an excellent condition. | yes, that is true | □1 | □2 | □3 | □4 | □5 | no, that is not true |

MFI® MULTIDIMENSIONAL FATIGUE INVENTORY

®E.Smets, B. Garssen, B. Bonke

**Reference** [17]: Smets EM, Garssen B, Bonke B, De Haes JC. The multidimensional fatigue inventory (MFI) psychometric qualities of an instrument to assess fatigue. J Psychosom Res 1995 Apr;39(3):315-325.

多軸向疲倦量表

| **說明：**  通過以下陳述，我們希望了解您最近的感覺。  以這個例子來說：「我感到輕鬆」。如果您認為這是完全正確的，您最近確實感到放鬆。那麼請你在最左邊的小格子劃X，就像這樣：  「**完全同意** ⮽1 □2 □3 □4 □5 **完全不正確**。」  您越不同意該語句，您就越可以將X放置在“完全不正確的”方向上。請不要錯過任何聲明，並且每個聲明只能在一個方框中放置一個X | | | | | | | |
| --- | --- | --- | --- | --- | --- | --- | --- |
| 01 | 我覺得我的體能很好。 **完全同意** | □1 | □2 | □3 | □4 | □5 | **完全不正確** |
| 02 | 體力上我覺得我只能做一點點事情。 **完全同意** | □1 | □2 | □3 | □4 | □5 | **完全不正確** |
| 03 | 我覺得我很活躍。 **完全同意** | □1 | □2 | □3 | □4 | □5 | **完全不正確** |
| 04 | 我覺得我想去做所有美好的事情。 **完全同意** | □1 | □2 | □3 | □4 | □5 | **完全不正確** |
| 05 | 我覺得我疲倦。 **完全同意** | □1 | □2 | □3 | □4 | □5 | **完全不正確** |
| 06 | 我覺得我一天能做許多事情。 **完全同意** | □1 | □2 | □3 | □4 | □5 | **完全不正確** |
| 07 | 當我在做事情時，我能專心於那件事情上。**完全同意** | □1 | □2 | □3 | □4 | □5 | **完全不正確** |
| 08 | 我的體力可以讓我從事許多事情。 **完全同意** | □1 | □2 | □3 | □4 | □5 | **完全不正確** |
| 09 | 我對要去處理事情感到畏懼。 **完全同意** | □1 | □2 | □3 | □4 | □5 | **完全不正確** |
| 10 | 我認為我一天只能做一點點事情。 **完全同意** | □1 | □2 | □3 | □4 | □5 | **完全不正確** |
| 11 | 我可以很專心。 **完全同意** | □1 | □2 | □3 | □4 | □5 | **完全不正確** |
| 12 | 我的休息是充份的。 **完全同意** | □1 | □2 | □3 | □4 | □5 | **完全不正確** |
| 13 | 我需要很努力才能專心在事情上。 **完全同意** | □1 | □2 | □3 | □4 | □5 | **完全不正確** |
| 14 | 在體能上，我覺得我處在一個很糟的狀態。**完全同意** | □1 | □2 | □3 | □4 | □5 | **完全不正確** |
| 15 | 我有許多的計畫。 **完全同意** | □1 | □2 | □3 | □4 | □5 | **完全不正確** |
| 16 | 我很容易感到疲倦。 **完全同意** | □1 | □2 | □3 | □4 | □5 | **完全不正確** |
| 17 | 我只能完成一點事情。 **完全同意** | □1 | □2 | □3 | □4 | □5 | **完全不正確** |
| 18 | 我不喜歡做任何事情。 **完全同意** | □1 | □2 | □3 | □4 | □5 | **完全不正確** |
| 19 | 我很容易恍神。  **完全同意** | □1 | □2 | □3 | □4 | □5 | **完全不正確** |
| 20 | 在體能上，我覺得我處在一個很好的狀態。**完全同意** | □1 | □2 | □3 | □4 | □5 | **完全不正確** |

**Reference** [18]: Chuang LL, Chuang YF, Hsu MJ, Huang YZ, Wong AMK, Chang YJ. Validity and reliability of the Traditional Chinese version of the Multidimensional Fatigue Inventory in general population. PLoS One. 2018 May 10;13(5):e0189850.
