# Supplementary material for: Applying a Smartwatch to Predict Work-related Fatigue for Emergency Healthcare Professionals: Machine Learning Method
Source: West J Emerg Med. 2023 Jul 7;24(4):693–702. doi: 10.5811/westjem.58139 (PMC10393460; doi:10.5811/westjem.58139)
Supplement: Supplementary file 2 [file wjem-24-693-s002.docx]

**Supplementary Table 2:** Characteristics and univariate analyses of variables (features) between medical personnel with or without fatigue on the training and testing cohorts.

|  | Training Cohort  (N=1079) | | P value | Testing Cohort  (N=463) | | P value |
| --- | --- | --- | --- | --- | --- | --- |
|  | Fatigue  (-)  (n=1022) | Fatigue  (+)  (n=57) |  | Fatigue  (-)  (n=435) | Fatigue  (+)  (n=28) |  |
| **Selected Features** | | | | | | |
| **Minimun of systolic pressure divided by diastolic pressure for the first 4-hour time interval, Mean (SD)** | 1.6 (0.1) | 1.6 (0.3) | 0.09 | 1.6 (0.1) | 1.4 (0.2) | <0.001 |
| **Minimun of systolic pressure divided by diastolic pressure for the last 4-hour time interval, Mean (SD)** | 1.6 (0.1) | 1.6 (0.3) | 0.05 | 1.6 (0.1) | 1.5 (0.3) | <0.001 |
| **Minimun of systolic pressure divided by diastolic pressure for the first 1-hour time interval, Mean (SD)** | 1.6 (0.1) | 1.7 (0.3) | 0.003 | 1.6 (0.1) | 1.4 (0.2) | <0.001 |
| **Minimun of systolic pressure divided by diastolic pressure for the last 1-hour time interval, Mean (SD)** | 1.6 (0.1) | 1.6 (0.2) | 0.28 | 1.6 (0.1) | 1.5 (0.2) | <0.001 |
| **Minimun of HRV for the first 1-hour time interval, Mean (SD)** | 27.7 (7.5) | 33.3 (9.0) | <0.001 | 28.4 (8.6) | 30.0 (9.1) | 0.39 |
| **Minimun of stress for the first 1-hour time interval, Mean (SD)** | 81.6 (6.7) | 85.4 (6.4) | 0.002 | 81.9 (7.1) | 82.8 (6.5) | 0.59 |
| **Maximum of systolic pressure divided by diastolic pressure for the last 1-hour time interval, Mean (SD)** | 1.6 (0.1) | 1.6 (0.2) | 0.69 | 1.6 (0.1) | 1.5 (0.2) | <0.001 |
| **Sum of calorie for the first 1-hour time interval, Mean (SD)** | 35.9 (22.5) | 31.0 (16.3) | 0.15 | 36.1 (22.6) | 31.4 (34.5) | 0.47 |
| **Work start time** |  |  | <0.001 |  |  | <0.001 |
| **7:00** | 53 (5.2) | 0 (0.0) |  | 7 (1.6) | 0 (0.0) |  |
| **7:30** | 409 (40.0) | 28 (49.1) |  | 126 (29.0) | 6 (21.4) |  |
| **8:00** | 29 (2.8) | 0 (0.0) |  | 15 (3.4) | 0 (0.0) |  |
| **9:00** | 7 (0.7) | 0 (0.0) |  | 6 (1.4) | 0 (0.0) |  |
| **14:30** | 44 (4.3) | 5 (8.8) |  | 8 (1.8) | 0 (0.0) |  |
| **15:30** | 455 (44.5) | 15 (26.3) |  | 202 (46.4) | 6 (21.4) |  |
| **20:00** | 8 (0.8) | 1 (1.8) |  | 16 (3.7) | 1 (3.6) |  |
| **23:30** | 17 (1.7) | 8 (14.0) |  | 55 (12.6) | 15 (53.6) |  |
| **Sum of calorie for the first 4-hour time interval, Mean (SD)** | 80.5 (40.8) | 75.9 (36.5) | 0.43 | 80.7 (44.0) | 69.3 (43.8) | 0.18 |
| **Sum of steps for the first 1-hour time interval, Mean (SD)** | 463.5 (290.0) | 489.0 (312.3) | 0.54 | 509.0 (304.4) | 515.0 (291.2) | 0.92 |
| **Minimun of heart rate for the first 1-hour time interval, Mean (SD)** | 69.4 (9.6) | 68.8 (8.2) | 0.68 | 69.0 (10.0) | 69.6 (11.4) | 0.79 |
| **Maximum of heart rate for the first 1-hour time interval, Mean (SD)** | 100.5 (14.0) | 99.4 (14.6) | 0.58 | 101.9 (14.1) | 102.9 (16.1) | 0.74 |
| **Standard deviation of heart rate for the first 1-hour time interval, Mean (SD)** | 11.9 (5.8) | 11.9 (4.9) | 0.96 | 12.6 (6.1) | 12.6 (6.3) | 0.99 |
| **Slope of linear regression of heart rate for the first 1-hour time interval, Mean (SD)** | 96.7 (253.1) | 112.0 (181.1) | 0.67 | 89.2 (283.0) | 42.5 (113.0) | 0.39 |
| **Maximum of heart rate for the first 4-hour time interval, Mean (SD)** | 108.9 (14.6) | 107.6 (13.3) | 0.52 | 109.9 (14.1) | 108.7 (15.9) | 0.66 |
| **Minimun of heart rate for the first 4-hour time interval, Mean (SD)** | 63.0 (6.9) | 62.7 (6.2) | 0.76 | 62.6 (6.8) | 62.5 (6.6) | 0.92 |
| **Standard deviation of stress for the first 4-hour time interval, Mean (SD)** | 4.5 (2.4) | 4.1 (2.1) | 0.43 | 4.5 (2.5) | 4.9 (2.2) | 0.43 |
| **Standard deviation of heart rate for the first 4-hour time interval, Mean (SD)** | 12.7 (4.3) | 12.6 (4.4) | 0.83 | 13.1 (4.4) | 13.1 (5.1) | 1 |
| **Slope of linear regression of stress for the first 4-hour time interval, Mean (SD)** | 70.7 (56.1) | 96.1 (33.4) | 0.02 | 75.1 (62.5) | 81.6 (33.8) | 0.63 |
| **Maximum of stress for the first 1-hour time interval, Mean (SD)** | 86.0 (6.0) | 88.2 (6.2) | 0.05 | 86.0 (6.0) | 87.2 (6.9) | 0.39 |
| **Slope of linear regression of heart rate for the first 4-hour time interval, Mean (SD)** | 101.5 (71.2) | 94.8 (78.0) | 0.51 | 97.4 (75.1) | 90.9 (39.0) | 0.65 |
| **Standard deviation of stress for the last 1-hour time interval, Mean (SD)** | 3.6 (2.6) | 3.6 (2.4) | 0.97 | 3.8 (2.7) | 2.8 (2.4) | 0.21 |
| **Standard deviation of HRV for the first 4-hour time interval, Mean (SD)** | 5.6 (3.0) | 5.8 (3.1) | 0.6 | 5.6 (3.2) | 7.2 (3.4) | 0.02 |
| **Slope of linear regression of systolic pressure divided by diastolic pressure for the first 4-hour time interval, Mean (SD)** | 1.6 (0.5) | 1.7 (0.4) | 0.35 | 1.6 (0.5) | 1.4 (0.4) | 0.04 |
| **Slope of linear regression of systolic pressure for the first 4-hour time interval, Mean (SD)** | 135.0 (59.0) | 124.3 (36.9) | 0.34 | 133.2 (50.8) | 113.1 (14.7) | 0.06 |
| **Standard deviation of HRV for the last 1-hour time interval, Mean (SD)** | 4.8 (3.5) | 4.9 (4.0) | 0.89 | 4.8 (3.5) | 5.0 (5.0) | 0.91 |
| **Sum of calorie for the last 4-hour time interval, Mean (SD)** | 53.9 (26.6) | 57.5 (26.8) | 0.33 | 58.9 (27.7) | 47.0 (28.7) | 0.03 |
| **Slope of linear regression of heart rate for the last 1-hour time interval, Mean (SD)** | 96.2 (438.0) | 161.7 (402.2) | 0.27 | 124.0 (408.2) | 110.3 (270.3) | 0.86 |
| **Standard deviation of heart rate for the last 1-hour time interval, Mean (SD)** | 12.1 (6.1) | 12.5 (5.9) | 0.57 | 11.7 (6.1) | 12.5 (5.1) | 0.53 |
| **Maximum of heart rate for the last 1-hour time interval, Mean (SD)** | 99.5 (15.1) | 100.7 (14.6) | 0.55 | 99.0 (14.9) | 98.9 (13.6) | 0.98 |
