# Supplementary material for: Applying a Smartwatch to Predict Work-related Fatigue for Emergency Healthcare Professionals: Machine Learning Method
Source: West J Emerg Med. 2023 Jul 7;24(4):693–702. doi: 10.5811/westjem.58139 (PMC10393460; doi:10.5811/westjem.58139)
Supplement: Supplementary file 4 [file wjem-24-693-s004.docx]

**Supplementary Table 4:** The description of the three types (demographics, sensor data from the smartwatch, and statistic data derived from the sensor data) of the candidate features.

1. Participants’ demographics

| metadata short name | meaning |
| --- | --- |
| age | age of the candidate (coarsely binned) |
| dayofweek | day of week of the day filling the form |
| work_starttime | time to start a work day |
| form_timeH_before | the time (hour) filling the form in the beginning of the work schedule |
| timedelta_after | the time difference between the second form filling time and the getting off time |
| timedelta_before | the time difference between the first form filling time and the work start time. |
| workhours | total work hours of the candidate at the day filling the forms |
| work_type | shifts of work schedule (daytime, night, midnight) |
| gender | gender of the candidate |
| role | work role of the candidate |
| room_after | the room where the candidate work at before getting off |
| room_before | the room where the candidate work at in the beginning of the work schedule |
| seniority | work years of the candidate (coarsely binned) |

2. Sensor data collected from the smartwatch

| Vital data short name | meaning | source |
| --- | --- | --- |
| hr | heart rate | vivowatch |
| sys | systolic blood pressure | vivowatch |
| dia | diastolic blood pressure | vivowatch |
| bp_minus | difference between two blood pressure: systolic - diastolic | derived |
| bp_ratio | ratio of blood pressures: systolic / diastolic | derived |
| bp_map | mean arterial pressure | derived |
| steps | walking steps | vivowatch |
| cals | calorie expenditure | vivowatch |
| rmssd | heart rate variability | vivowatch |
| stress | stress index calculated by vivowatch | vivowatch |
| shockindex | ratio between systolic blood pressure and heart rate | derived |

3. The top 30 Features derived from the statistics of the sensor data, plus one feature (work start time) from the demographics of the participants,

| feature name | Entropy measure | measurements | time period | statistical function |
| --- | --- | --- | --- | --- |
| h4p0_bp_divide_min | 0.036543 | ratio of blood pressure (sys/dia) | first 4 hours | minimum |
| h4m1_bp_divide_min | 0.036137 | ratio of blood pressure (sys/dia) | last 4 hours | minimum |
| h1p0_bp_divide_min | 0.034233 | ratio of blood pressure (sys/dia) | first 1 hour | minimum |
| h1m1_bp_divide_min | 0.028627 | ratio of blood pressure (sys/dia) | last 1 hour | minimum |
| h1p0_rmssd_min | 0.015196 | heart rate variability | first 1 hour | minimum |
| h1p0_stress_min | 0.014787 | stress index | first 1 hour | minimum |
| h1m1_bp_divide_max | 0.014614 | ratio of blood pressure (sys/dia) | last 1 hour | maximum |
| h1p0_cal_sum | 0.012602 | calorie expenditure | first 1 hour | summation |
| work_starttime | 0.012323 | time to start a work day |  |  |
| h4p0_cal_sum | 0.008767 | calorie expenditure | first 4 hours | summation |
| h1p0_step_sum | 0.006543 | steps | first 1 hour | summation |
| h1p0_hr_min | 0.006266 | heart rate | first 1 hour | minimum |
| h1p0_hr_max | 0.006266 | heart rate | first 1 hour | maximum |
| h1p0_hr_std | 0.005753 | heart rate | first 1 hour | standard deviation |
| h1p0_hr_poly1slope | 0.004671 | heart rate | first 4 hours | the slope of linear fit |
| h4p0_hr_max | 0.003619 | heart rate | first 4 hours | maximum |
| h4p0_hr_min | 0.003619 | heart rate | first 4 hours | minimum |
| h4p0_stress_std | 0.003592 | stress | first 4 hours | standard deviation |
| h4p0_hr_std | 0.003397 | heart rate | first 4 hours | standard deviation |
| h4p0_stress_poly1slope | 0.003357 | stress | first 4 hours | the slope of linear fit |
| h1p0_stress_max | 0.003337 | stress | first 1 hour | maximum |
| h4p0_hr_poly1slope | 0.00319 | heart rate | first 4 hours | the slope of linear fit |
| h1m1_stress_std | 0.002954 | stress index | last 1 hour | standard deviation |
| h4p0_rmssd_std | 0.00285 | heart rate variability | first 4 hours | standard deviation |
| h4p0_bp_divide_poly1slope | 0.002831 | ratio of blood pressure (sys/dia) | first 4 hours | the slope of linear fit |
| h4p0_sys_poly1slope | 0.002831 | blood pressure (sys) | first 4 hours | the slope of linear fit |
| h1m1_rmssd_std | 0.001955 | heart rate variability | last 1 hour | standard deviation |
| h4m1_cal_sum | 0.001384 | calorie expenditure | last 4 hour | summation |
| h1m1_hr_poly1slope | 0.000921 | heart rate | last 1 hour | the slope of linear fit |
| h1m1_hr_std | 0.000566 | heart rate | last 1 hour | standard deviation |
| h1m1_hr_max | 0.000525 | heart rate | last 1 hour | maximum |
